# Measurement of myocardial blood flow by cardiovascular magnetic resonance perfusion: comparison of distributed parameter and Fermi models with single and dual bolus

**DOI:** 10.1186/s12968-015-0125-1

**Published:** 2015-02-17

**Authors:** Giorgos Papanastasiou, Michelle C Williams, Lucy E Kershaw, Marc R Dweck, Shirjel Alam, Saeed Mirsadraee, Martin Connell, Calum Gray, Tom MacGillivray, David E Newby, Scott IK Semple

**Affiliations:** Clinical Research Imaging Centre, University of Edinburgh,, Edinburgh, UK; Centre for Cardiovascular Science, University of Edinburgh, Edinburgh, UK; Christie Medical Physics and Engineering, The Christie NHS FT, Manchester, UK

**Keywords:** Cardiovascular magnetic resonance, Myocardial blood flow, Fermi modeling, Distributed parameter modeling, Fractional flow reserve, Invasive coronary angiography

## Abstract

**Background:**

Mathematical modeling of cardiovascular magnetic resonance perfusion data allows absolute quantification of myocardial blood flow. Saturation of left ventricle signal during standard contrast administration can compromise the input function used when applying these models. This saturation effect is evident during application of standard Fermi models in single bolus perfusion data. Dual bolus injection protocols have been suggested to eliminate saturation but are much less practical in the clinical setting. The distributed parameter model can also be used for absolute quantification but has not been applied in patients with coronary artery disease. We assessed whether distributed parameter modeling might be less dependent on arterial input function saturation than Fermi modeling in healthy volunteers. We validated the accuracy of each model in detecting reduced myocardial blood flow in stenotic vessels versus gold-standard invasive methods.

**Methods:**

Eight healthy subjects were scanned using a dual bolus cardiac perfusion protocol at 3T. We performed both single and dual bolus analysis of these data using the distributed parameter and Fermi models. For the dual bolus analysis, a scaled pre-bolus arterial input function was used. In single bolus analysis, the arterial input function was extracted from the main bolus. We also performed analysis using both models of single bolus data obtained from five patients with coronary artery disease and findings were compared against independent invasive coronary angiography and fractional flow reserve. Statistical significance was defined as two-sided P value < 0.05.

**Results:**

Fermi models overestimated myocardial blood flow in healthy volunteers due to arterial input function saturation in single bolus analysis compared to dual bolus analysis (P < 0.05). No difference was observed in these volunteers when applying distributed parameter-myocardial blood flow between single and dual bolus analysis. In patients, distributed parameter modeling was able to detect reduced myocardial blood flow at stress (<2.5 mL/min/mL of tissue) in all 12 stenotic vessels compared to only 9 for Fermi modeling.

**Conclusions:**

Comparison of single bolus versus dual bolus values suggests that distributed parameter modeling is less dependent on arterial input function saturation than Fermi modeling. Distributed parameter modeling showed excellent accuracy in detecting reduced myocardial blood flow in all stenotic vessels.

**Electronic supplementary material:**

The online version of this article (doi:10.1186/s12968-015-0125-1) contains supplementary material, which is available to authorized users.

## Background

Mathematical modeling of cardiovascular magnetic resonance perfusion (CMR) has the potential to allow quantitative assessment of myocardial blood flow [1,2]. Establishing absolute quantification of blood flow could have clinical benefits since it may lead to an improvement in the diagnosis and prognostication of patients with coronary artery disease [3-6].

Myocardial blood flow quantification using model-dependent analysis is based on fitting the convolution of a model with the arterial input function to the tissue contrast agent concentration-time curve. The model describes the passage of a contrast agent through the myocardium whilst the arterial input function is the observed contrast agent concentration-time curve derived from the blood pool [3]. Fermi deconvolution modeling is a popular approach used to estimate myocardial blood flow during the first-pass of gadolinium-based extracellular contrast agents (CA) [3,7,8]. It is an empirical-mathematical model, which is convolved with the first-pass of the arterial input function [3]. The distributed parameter model assumes that the extravascular-extracellular space exchanges CA with nearby regions in the intravascular space, restricting axial transport of CA inside the extravascular-extracellular space [4]. In addition to myocardial blood flow, this model can also be used to calculate other microvascular characteristics including intravascular space, extravascular-extracellular space, permeability surface area product, extraction fraction and volume of distribution [9].

The high concentration of CA during bolus passage leads to signal saturation and causes concentration underestimation in the left ventricular cavity [10] (which is used to generate an arterial input function for model deconvolution analysis). This can degrade the accuracy and reproducibility of myocardial blood flow quantification using Fermi modeling, leading to systematic myocardial blood flow overestimation [3]. The dual bolus acquisition technique can eliminate signal saturation allowing more reliable quantification of myocardial blood flow in first-pass magnetic resonance perfusion imaging. In the dual bolus technique, an initial injection of dilute CA is used to acquire a non-saturated arterial input function before the main CA bolus. This is commonly referred to as the “pre-bolus” [11,12]. However, compared to single bolus protocols [1-3,7-9], dual bolus imaging protocols are characterized by increased complexity both in image acquisition and data analysis [3,10-12].

In the present study, we compared single and dual bolus estimates of myocardial blood flow in healthy volunteers using both distributed parameter and Fermi models. We also assessed whether these models can reliably detect areas with reduced myocardial blood flow compared to a clinical gold standard of invasive coronary angiography and fractional flow reserve in patients with coronary artery disease.

## Methods

### Study population

Eight healthy volunteers with no previous history of cardiovascular or renal disease, diabetes mellitus, asthma or any other clinically significant illness and five patients with suspected coronary artery disease were recruited into the study. Exclusion criteria included severely compromised renal function (estimated glomerular filtration rate <30 mL/min) and contraindications to magnetic resonance imaging. The study was performed with the approval of the local research ethics committee, in accordance with the Declaration of Helsinki and with the written informed consent of all subjects. Prior to CMR perfusion, all subjects were asked to refrain from caffeine for 12 hours.

### Image acquisition

All data were acquired using a 3T Verio system (Siemens AG, Healthcare Sector, Erlangen, Germany). Standard cardiac imaging planes and a short axis stack of left ventricular cine data were acquired using routine steady state free precession (TrueFISP) acquisitions. Native T_1_ relaxation rates (i.e. in the absence of CA) were calculated using the modified Look-Locker inversion (MOLLI) recovery technique [13]. Stress imaging was performed by intravenous infusion of 140 μg/kg/min of adenosine (Adenoscan, Sanofi Aventis). Fifty dynamic perfusion images were obtained at diastole across three short-axis view slices: basal, mid-ventricular and apical slices according to the standard 16-segment heart model [14]. Perfusion images were acquired using a turbo-fast low angle shot (FLASH) saturation recovery prepared single-shot gradient echo pulse sequence (repetition time/ echo time 2.20 ms/1.07 ms, flip angle 12^o^, slice thickness 8 mm, preparation pulse delay (PD) to central line of k-space 100 ms, matrix size 192 × 108 and FoV 330 mm × 440 mm). With the application of GRAPPA (accelerator factor of 3) and partial Fourier acquisition of 0.75, each dynamic frame consisted of 48-phase encoded lines. All CMR perfusion data were acquired using electrocardiogram-gating.

### Contrast agent bolus administration

In single bolus imaging, 0.05 mmol/kg of CA (Gadovist, Bayer Healthcare) was injected intravenously after 4 min of adenosine infusion, followed by 20 mL of 0.9% saline (Medrad Spectris Solaris, Medrad, USA) at 4 mL/s [3]. All patients with coronary artery disease were imaged using the single bolus protocol.

In the healthy volunteer cohort, an additional pre-bolus infusion was administered to allow dual bolus modeling to be applied without the risk of signal saturation in the arterial input function. In this dual bolus protocol, the two boluses were injected in a pre-determined concentration ratio (pre-bolus:main bolus, 1:5) with the pre-bolus diluted using 0.9% saline. After 3.5 min of adenosine infusion, the pre-bolus of 0.006 mmol/kg CA was injected and adenosine was continued until the main bolus of 0.03 mmol/kg had also been administered. The pre-bolus allows determination of the arterial input function whilst the main bolus allows measurement of myocardial CA concentration curves [15]. To allow clearance of residual CA, the rest perfusion imaging was performed 15 min after the adenosine-stress scan with the same acquisition protocol in all subjects [3,7,8,15].

### Invasive coronary angiography and fractional flow reserve

All five patients underwent invasive coronary angiography at the Royal Infirmary of Edinburgh. Fractional flow reserve was assessed for major epicardial vessels and defined as the ratio between distal coronary pressure and aortic pressure measured simultaneously at maximal adenosine-induced (intravenous 140μg/kg/min) hyperaemia [16,17]. Haemodynamically significant coronary artery disease was defined as luminal stenosis ≥70% on invasive coronary angiography, or fractional flow reserve < 0.80 and luminal stenosis ≥50 %. Outcomes from the three main coronary vessels were classified into 3 groups: Group 1, no or minor coronary artery disease with luminal stenosis <50%; Group 2, non-obstructive coronary artery disease with luminal stenosis ≥50% and fractional flow reserve > 0.80; and Group 3, obstructive coronary artery disease with luminal stenosis of ≥70% alone, or luminal stenosis ≥50% and fractional flow reserve ≤ 0.80 [16,17].

### Cardiac contouring

Endocardial and epicardial contours were manually defined on the short axis magnetic resonance perfusion imaging data using dedicated cardiac image analysis software (QMass, Medis, The Netherlands) to generate a standardised 16-segment model of the heart [14]. Myocardial blood flow analysis was performed per myocardial segment. The signal intensity of the arterial input function was extracted from the left ventricular cavity excluding papillary muscles using customised in-house software created in Matlab (MathWorks Inc., Natick, MA) [18].

All arterial input function curves were extracted from the basal slice [3,7]. In single bolus analysis, the arterial input function was extracted from the standard (main bolus) CA dose component. For the dual bolus analysis in healthy subjects, the pre-bolus arterial input function was scaled and used for deconvolution analysis [3,11,15].

### Image processing

To correct for signal saturation, myocardial and arterial input function signal intensity-time curves were converted to CA concentration-time curves using the method of Larsson *et al* [19], as described previously [3,7-9,11,12,19,20]. This method is based on the assumption that in a region of interest, the longitudinal relaxation rate R_1_ (1/T_1_) changes linearly as a function of contrast agent concentration influx c(t) at time t multiplied by its relaxivity r_1_ according to the following equation:

1 $$ \frac{1}{{\mathrm{T}}_1\left(\mathrm{t}\right)}-\frac{1}{{\mathrm{T}}_1(0)}=r1\cdot c(t), $$

where T_1_(0) is the native longitudinal relaxation rate and T_1_(t) is the longitudinal relaxation rate at time t of contrast enhancement. By substituting $\varDelta {R}_1\frac{1}{{\mathrm{T}}_1\left(\mathrm{t}\right)}-\frac{1}{{\mathrm{T}}_1(0)},$ equation (1) can be re-written as:

2 $$ c(t)=\frac{\varDelta {R}_1}{r_1} $$

In the above set of equations, T_1_(t) is unknown and can be calculated by adapting the MR signal equation for the saturation recovery prepared single-shot FLASH sequence as follows [3,7,19]:

3 $$ SI=\uppsi \cdot \left[\left(1-{e}^{-PD\cdot {R}_1}\right)\cdot {a}^{n-1}+b\cdot \frac{1-a}{1-a}\right] $$

where SI is the signal intensity, Ψ is a calibration constant dependent on receiver gain, instrumental conditions, proton density and α. PD is the pre-pulse delay which is the time between saturation pulse and the central line of k-space, $a= \cos \left(\alpha \right)\cdot {e}^{-TR\cdot {R}_1}$ and $b=1-{e}^{-TR\cdot {R}_1}$ . TR is the time interval between repetitive α-radiofrequency pulses. Ψ is assumed to be constant throughout the dynamic perfusion image acquisition [19] and was initially calculated from equation (3) by using native T_1_ and signal intensities derived from a region of interest (i.e. myocardial segment, arterial input function) in the absence of CA. T_1_(t) at time t of contrast enhancement was then calculated from equation (3) using Ψ and signal intensity values extracted from the same region of interest in each of the dynamic perfusion images. CA concentration-time curves were then calculated using equation (2).

### Model equations

The model equations used for data fitting are summarized in Table 1. These equations represent the tissue impulse response R(t) the shape of which is determined by the fitted parameters [3]. To quantify myocardial blood flow and other parameters generated by the Fermi and distributed parameter models, we used model-constrained deconvolution [3,7,9]. The Fermi model was fitted in the time domain whilst the distributed parameter model was fitted in the Laplace domain in order to avoid discontinuities of the time step-function that can be present when fitting the distributed parameter model in the time domain [9,21]. We fitted the convolution of the Fermi function with the first-pass of the arterial input function, setting the end-point at the CA concentration minimum before the recirculation component begins (this range varies from patient to patient but is commonly in the range between 20-35 dynamic frames). We also fitted the convolution of the distributed parameter function with the entire CA concentration time course of the arterial input function (i.e. 50 dynamic frames per slice). To further investigate the behaviour of distributed parameter modeling in single and dual bolus analysis, we also fitted the convolution of the distributed parameter model with the first-pass of the arterial input function using the same number of time points as in Fermi modeling. All additional microvascular parameters were calculated using the relationships described in Table 2 [4,9].

**Table 1:** **Model equations**

**Model**	**Fitted parameters**	**Fitting domain**	**Tissue impulse response** ***R***
Distributed parameter	Myocardial blood flow, T, T_c_, T_e_	Laplace	$R(s)=\frac{1- \exp \left[-s\cdot \left(T+s\cdot {T}_c\cdot {T}_e\right)/\left(1+s\cdot {T}_e\right)\right]}{s}$
Fermi	Myocardial blood flow, τ_0,_ k	Time	$R(t)=\frac{1}{ \exp \left[\left(t-{\tau}_0\right)\cdot k\right]+1}$

Fitted parameters for distributed parameter: myocardial blood flow, T is mean overall transit time, T_c_ is mean capillary transit time, T_e_ is mean interstitial (i.e. extravascular-extracellular) transit time. Where *s* = *i* ⋅ 2 ⋅ *π* ⋅ *f* and *f* is the frequency variable in the Fourier transformed data. Fitted parameters for Fermi: myocardial blood flow, τ_0_ characterized the width of the shoulder of the Fermi function and k determined the decay rate of R(t) due to contrast agent wash-out. t is the time variable.

**Table 2:** **Microvascular characteristics**

**Microvascular characteristics**	**Equation**
*v*_*b*_	*v*_*b*_ = *MBF* ⋅ *T*_*c*_
*v*_*e*_	*v*_*e*_ = *MBF* ⋅ (*T* − *T*_*c*_)
*v*_*d*_	*v*_*d*_ = *MBF* ⋅ *T*
*PS*	$PS=\frac{MBF\cdot \left(T-{T}_c\right)}{T_e}$
*E*	$E=1- \exp \left(-\frac{PS}{MPF}\right)$
*MPF*	*MPF* = *MBF* ⋅ (1 − *hct*)

Microvascular characteristics were calculated by incorporating the fitted parameters of the distributed parameter model into the following relationships (see reference [4]). Myocardial plasma flow (MPF) was used to calculate extravascular-extracellular space (v_e_), distribution volume (v_d_), permeability surface area product (PS) and extraction fraction (E) and myocardial blood flow (MBF) to calculate intravascular space (v_b_). Hematocrit: hct.

A haematocrit value of 0.45 was assumed in order to convert myocardial blood flow into plasma flow which was used to calculate permeability surface area product, extraction fraction, extravascular-extracellular space and volume of distribution. Both models were fitted using a constrained nonlinear optimization (fmincon) in Matlab [22]. Myocardial perfusion reserve (myocardial blood flow at stress/ myocardial blood flow at rest) was calculated for all healthy volunteer data. Consistent with previous cardiac perfusion studies, vessel territories in patients with hyperaemic myocardial blood flow values less than 2.5 mL/min/mL of tissue were considered as regions with reduced myocardial blood flow [5,6].

### Statistical analysis

The R software was used for statistical analysis (R Foundation for statistical computing, Vienna, Austria). Identification of any systematic bias between dual bolus and single bolus modeling estimates was performed using Bland Altman plots for both models. Statistical differences were investigated between Fermi and distributed parameter modeling, between distributed parameter and first-pass distributed parameter modeling, between stress and rest modeling values as well as between dual and single bolus analysis by implementing a paired *t*-test. A Welch two sample *t*-test was used to investigate statistical differences in myocardial blood flow values between the different groups (Groups 1-3) classified at the time of invasive coronary angiography.

Homogeneity of variances was verified using a Fisher’s F-test. Comparison of mean myocardial blood flow and physiological parameters estimates in vessel territories of patients versus overall mean values in healthy volunteers was investigated using one sample *t*-test. Statistical significance was defined as two-sided P value < 0.05.

## Results

**Figure 1:**
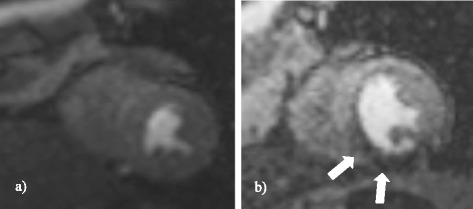
**Mid-ventricular dynamic CMR perfusion images are shown.** CMR perfusion image from **a)** a healthy volunteer and **b)** a patient with a perfusion abnormality in the infero-septal and inferior myocardial regions (white arrows).

The distributed parameter model was fitted in 8 healthy volunteers and 5 patients with coronary artery disease. Example images are shown in Figure 1. We generated 416 CA concentration-time curves in 13 subjects (16 myocardial segments per subject both at stress and rest). Distributed parameter model fits were successful in 398 CA concentration-time curves and non-convergent in 7 myocardial segments of one volunteer at stress, in 5 myocardial segments of one volunteer at rest and in 6 segments of one patient at stress. The Fermi model successfully fitted all CA concentration-time courses.

### Comparison of Fermi and distributed parameter models in healthy volunteers

**Figure 2:**
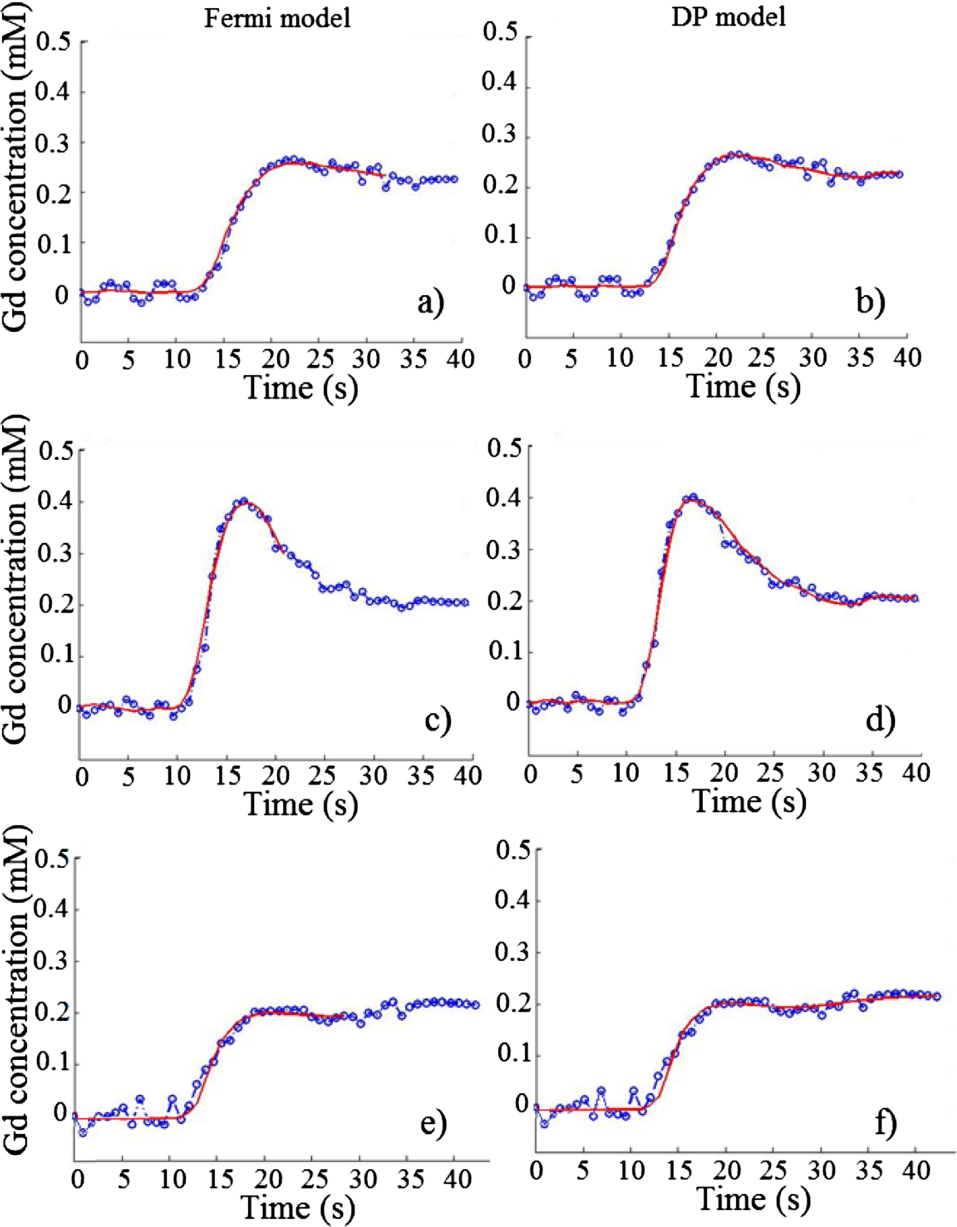
**Examples of Fermi and distributed parameter model fits.** Examples of model fits at rest **(a, b)** and at stress **(c, d)** from the same volunteer (dual bolus analysis). Fermi **(e)** and distributed parameter **(f)** model fits during hyperemia of a pathological myocardial segment (single bolus analysis). DP:distributed parameter model, Gd: gadolinium.

**Figure 3:**
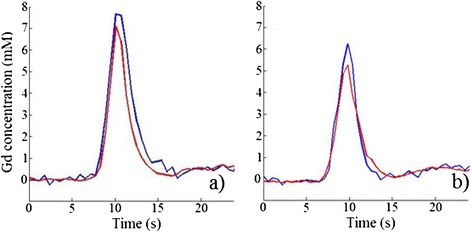
**Scaled pre-bolus arterial input function versus standard arterial input function from the same examination.** In volunteer 1 **(a)** and volunteer 2 **(b)** scaled pre-bolus (blue) arterial input function and main bolus arterial input function (red) are shown. Gd: gadolinium.

We initially fitted the Fermi and distributed parameter models to CA concentration-time curves for our healthy volunteer population using arterial input functions derived from the main bolus data. Examples of Fermi and distributed parameter model fits at rest and stress are presented in Figure 2. Examples of pre-bolus and main bolus arterial input functions are shown in Figure 3. Fermi-derived myocardial blood flow values were higher than distributed parameter-derived myocardial blood flow values for both stress and rest (P = 0.0005 and P = 0.007 respectively, Table 3).

**Table 3:** **Healthy volunteer mean (SD) myocardial blood flow values calculated using dual and single bolus analysis**

**Modeling values/Method**	**Fermi**	**Fermi**	**DP**	**DP**	**DP-First pass**	**DP-First pass**
	**Dual bolus**	**Single bolus**	**Dual bolus**	**Single bolus**	**Dual bolus**	**Single bolus**
Myocardial blood flow-Stress (mL/min/mL)	3.57 (0.59)*	4.57 (0.62)*	3.16 (0.71)	3.45 (0.48)	3.39 (0.56)	3.47 (0.50)
Myocardial blood flow-Rest (mL/min/mL)	1.48 (0.40)	1.57 (0.33)	1.23 (0.26)	1.46 (0.29)	1.18 (0.26)	1.34 (0.31)

Statistical differences between single and dual bolus analysis are indicated with *.

We subsequently fitted the Fermi and distributed parameter models for our healthy volunteer population, using scaled arterial input functions from their pre-bolus data. Fermi-derived myocardial blood flow values were again higher than distributed parameter-derived myocardial blood flow values for both stress and rest (P = 0.03 and P = 0.003 respectively, Table 3).

Mean distributed parameter model-derived myocardial blood flow at stress was not different in dual bolus compared to single bolus analysis (P = 0.22) whilst mean Fermi model-derived myocardial blood flow at stress was higher in single bolus versus dual bolus analysis (P = 0.00003, Table 3).

Systematic bias of the above comparisons was investigated using Bland Altman method. The average bias was computed as the blood flow values at stress determined in dual bolus minus the relative values determined in the single bolus analysis. For the Fermi model, the average bias was -1.00 with 95% confidence intervals [-1.58, -0.42] and for the distributed parameter model, the average bias value was -0.30 with 95% confidence intervals [-1.61, 0.94].

Mean Fermi and distributed parameter-derived myocardial blood flow at rest did not significantly change between single and dual bolus analysis (P = 0.07 for both). The additional distributed parameter estimates were not significantly different in single bolus compared to dual bolus analysis (see values in Additional file 1).

Mean myocardial blood flow was higher during hyperaemia in all healthy volunteers for distributed parameter-dual bolus (P = 0.00001), Fermi-dual bolus (P = 0.0000001), distributed parameter-single bolus (P = 0.0000005), and Fermi-single bolus analysis (P < 0.0000001). Mean (SD) myocardial perfusion reserve values were: 2.59 (0.37) for distributed parameter-dual bolus, 2.42 (0.30) for distributed parameter-single bolus, 2.51 (0.48) for Fermi-dual bolus and 2.96 (0.34) for Fermi-single bolus analysis.

To investigate the lack of dependence of the distributed parameter model to arterial input function saturation observed in single bolus data, we also performed first-pass distributed parameter modeling. There was no difference between distributed parameter and first pass distributed parameter myocardial blood flow values (P = 0.17 in dual bolus, P = 0.79 in single bolus analysis, Table 3). No difference was observed in first-pass distributed parameter-derived myocardial blood flow values between single and dual bolus analysis, for both stress (P = 0.31) and rest (P = 0.16) (Table 3).

### Distributed parameter and Fermi analysis in patients with coronary artery disease

Invasive coronary angiography and fractional flow reserve identified 7 vessels with obstructive lesions (Group 3), 5 vessels with non-obstructive lesions (Group 2) and 3 vessels with no or minor coronary artery disease (Group 1).

**Figure 4:**
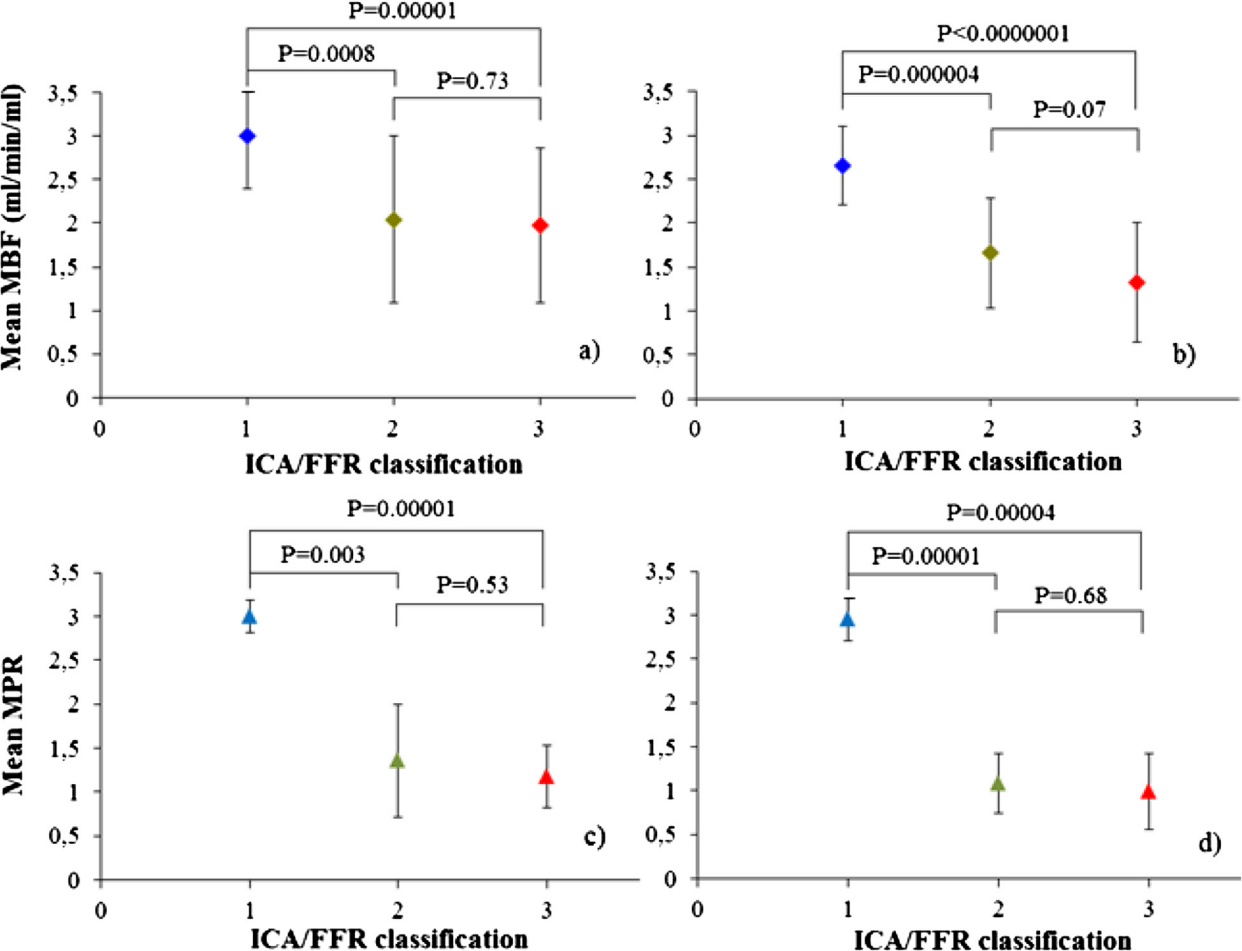
**Mean Fermi-MBF (a), distributed parameter-MBF (b), Fermi-MPR (c), distributed parameter MPR (d) versus ICA/FFR classification.** MBF: myocardial blood flow, MPR: myocardial perfusion reserve, ICA: invasive coronary angiography, FFR: fractional flow reserve.

Mean myocardial blood flow values were calculated in vessel territories of the three main coronary arteries for each patient using both models (Table 4, Figure 2e and f). The Fermi and distributed parameter models correctly identified reduced myocardial blood flow in 6 and 7 of the 7 vessels in Group 3 respectively. In addition, the Fermi and distributed parameter models correctly identified reduced myocardial blood flow in 3 and 5 of the 5 vessels in Group 2 respectively. Both models estimated myocardial blood flow within normal range in Group 1. A difference was observed in myocardial blood flow at stress and in myocardial perfusion reserve between Group 1 versus Groups 2 and 3 for both models (Figure 4, Table 4).

**Table 4:** **Invasive coronary angiography/fractional flow reserve classification and mean myocardial blood flow (SD) at stress measured in mL/min/mL per vessel territories of the three main coronary arteries**

		**Invasive coronary angiography/Fractional flow reserve**	**Distributed parameter-Myocardial blood flow**	**Fermi-Myocardial blood flow**	**Distributed parameter-Myocardial perfusion reserve**	**Fermi-Myocardial perfusion reserve**
Patient 1	LAD	3	0.82 (0.28)*	1.68 (0.60)*	0.88 (0.29)	1.54 (0.47)
	LCX	3	0.94 (0.20)*	1.99 (0.41)*	0.91 (0.16)	1.73 (0.44)
	RCA	2	0.84 (0.17)*	1.77 (0.79)*	0.91 (0.24)	1.77 (0.44)
Patient 2	LAD	2	1.99 (0.30)*	3.37 (0.49)	1.68 (0.37)	2.15 (0.49)
	LCX	2	1.98 (0.27)*	2.61 (0.41)	1.26 (0.30)	1.87 (0.80)
	RCA	3	1.27 (0.27)*	1.80 (0.81)*	0.86 (0.30)	1.08 (0.33)
Patient 3	LAD	2	1.20 (0.10)*	1.19 (0.34)*	0.71 (0.11)	0.78 (0.44)
	LCX	3	1.34 (0.13)*	1.84 (1.11)*	0.65 (0.30)	0.96 (0.26)
	RCA	2	1.58 (0.31)*	1.18 (0.16)*	0.81 (0.20)	0.70 (0.12)
Patient 4	LAD	3	1.99 (0.31)*	3.02 (0.64)	1.21 (0.31)	1.22 (0.23)
	LCX	3	1.61 (0.73)*	1.98 (0.58)*	0.90 (0.35)	1.05 (0.34)
	RCA	3	0.75 (0.29)*	1.00 (0.44)*	0.58 (0.23)	0.65 (0.24)
Patient 5	LAD	1	2.86 (0.59)	3.26 (0.88)	3.26 (0.40)	3.37 (0.50)
	LCX	1	2.54 (0.24)	2.79 (0.30)	3.01 (0.60)	2.91 (0.34)
	RCA	1	2.60 (0.36)	2.88 (0.33)	2.68 (0.35)	3.04 (0.85)

LAD, LCX and RCA: left anterior descending, left circumflex and right coronary artery respectively. Vessels with reduced myocardial blood flow are indicated with *.

Mean physiological parameter values were also calculated using distributed parameter modeling in all vessel territories for all patients (see Additional file 1).

## Discussion

We have compared single and dual bolus estimates of myocardial perfusion in healthy volunteers using both Fermi and 2-region 1-barrier distributed parameter models. We demonstrate no difference in myocardial blood flow estimates using the distributed parameter model between single and dual bolus analysis. In agreement with previous work, we demonstrate an increase in stress myocardial blood flow estimates with application of Fermi modeling using single bolus data analysis compared to dual bolus data analysis. For the first time, we have also successfully fitted the distributed parameter model in patients with coronary artery disease.

### Model comparison in healthy volunteers

Using the distributed parameter model, we successfully fitted 96% of our data (398 in 416 CA concentration-time courses). Model comparison in 8 healthy volunteers suggested that single bolus analysis of the distributed parameter model shows no statistically significant difference compared to dual bolus analysis, indicating that this model may be less dependent on arterial input function saturation than the Fermi model. Furthermore, distributed parameter modeling using the first pass only, showed no statistically significant difference between single bolus and dual bolus analysis. This shows that the lack of dependence on single or dual bolus in the distributed parameter model using the full curve is not due to the increased number of time points used for fitting, compared to the first pass Fermi model. Dual bolus [11,12,15,23] and dual sequence [10,24] (which includes a low resolution dynamic acquisition of the left ventricle to eliminate arterial input function saturation), are the most widely suggested techniques to solve the issue of arterial input function saturation. However, both of these techniques involve increased complexity in image acquisition and data analysis that have led to ongoing debate regarding whether either method might replace standard single bolus protocols for CMR perfusion and myocardial blood flow quantification. Whilst single bolus protocols are prone to arterial input function saturation, they are still widely used in clinical imaging and are suitable for qualitative assessment of myocardial perfusion. Our analysis suggests that peak arterial input function saturation may not be such a dominant factor when quantifying myocardial blood flow in distributed parameter modeling compared with Fermi modeling.

Our calculated myocardial blood flow and microvascular characteristic parameter values generally agree with a previous study that was the first to introduce the two-region, one-barrier distributed parameter model in cardiac data [9]. Broadbent *et al* fitted a distributed parameter model in data acquired using a different protocol: short-axis view of the entire myocardial area across one mid-ventricular slice acquired in systole at 1.5 T. We applied the distributed parameter model using a 16-segment heart model across three mid-ventricular slices acquired in diastole at 3T.

### The impact of contrast agent dose

We validated the dependence of Fermi and distributed parameter modeling in the presence of arterial input function saturation in single bolus data, using a relatively low CA dose (0.03 mmol/kg) in our healthy volunteer cohort. The administration of the specific CA dose has possibly caused limited arterial input function saturation at the peak of contrast enhancement [3,10] (as shown in Figure 3), compared to higher CA doses. Our study demonstrates that Fermi modeling is still sensitive to any arterial input function saturation present in our single bolus data. In contrast, distributed parameter modeling is less dependent on any arterial input function saturation present in our data. Any increases in CA dose (at 3T), can increase the degree of arterial input function saturation in single bolus data of healthy volunteers, which would necessitate a de novo validation of distributed parameter modeling in single against dual bolus analysis.

### Distributed parameter and Fermi analysis in patients

The distributed parameter model was capable of detecting reduced myocardial blood flow in patients with non-obstructive and obstructive coronary artery disease (Groups 2 and 3 respectively). Distributed parameter modeling correctly identified all 7 obstructive lesions and all 5 non-obstructive lesions. Fermi modeling correctly identified 6 out of 7 obstructive lesions and 3 out of 5 non-obstructive lesions. Both models showed decreased myocardial blood flow values as a function of luminal stenosis severity against invasive coronary angiography and fractional flow reserve classification (Figure 4).

### Study limitations

The number of subjects included in this study is small. However, this is the first study demonstrating that a 1-barrier 2-region distributed parameter model approach may be less dependent on arterial input function saturation than Fermi modeling. Distributed parameter modeling needs to be applied in larger patient cohorts to further validate its diagnostic accuracy. We have not validated the behaviour of distributed parameter modeling in higher CA doses. To reduce patient discomfort during administration of adenosine, we did not implement a dual bolus stress-rest protocol in our patient cohort. As such, it was impossible to validate any systematic errors that may have contaminated our patient myocardial blood flow quantifications due to arterial input function saturation. To overcome this limitation and to complement our model comparison, we further assessed the ability of both models in detecting reduced myocardial blood flow in stenotic vessels versus current invasive gold standard methods. The CA dose used in patients was higher than in healthy volunteers to increase the signal-to-noise ratio due to an assumed reduction in blood flow in our patient cohort as compared to our healthy volunteer cohort. Although this higher dose in patients has possibly caused some myocardial blood flow overestimations in Fermi modeling (Table 4, in two epicardial vessels in patient 2, and one epicardial vessel in patient 4), the distributed parameter modeling showed excellent accuracy in detecting reduced haemodynamics at stress in all stenotic vessels compared to our invasive gold standard. The vessels with non-obstructive disease (Group 2) were all from patients who also had one or two other vessels with obstructive disease (Group 3). The coincidental effect of microvascular dysfunction could therefore explain the low myocardial blood flow measurements in Group 2.

## Conclusions

We implemented a two-region, one-barrier distributed parameter model in healthy volunteers and patients with coronary artery disease. Distributed parameter-derived myocardial blood flow did not significantly change when a single bolus arterial input function was used compared to the dual bolus case. Fermi modelling of the same data demonstrated significant overestimations in myocardial blood flow in single bolus compared to dual bolus analysis. This suggests that the distributed parameter model might be less dependent on arterial input function saturation than Fermi modeling.

The distributed parameter model detected reduced myocardial blood flow in all 7 vessels with obstructive lesions and in all 5 vessels with non-obstructive lesions as determined by invasive coronary angiography and fractional flow reserve classification in a pilot cohort of 5 patients with coronary artery disease.

## Additional file

Additional file 1:
**Mean microvascular characteristics (SD) estimates for healthy volunteers and for all 3 invasive coronary angiography/fractional flow reserve Groups.**
