# Supplementary material for: Measurement of myocardial blood flow by cardiovascular magnetic resonance perfusion: comparison of distributed parameter and Fermi models with single and dual bolus
Source: J Cardiovasc Magn Reson. 2015 Feb 17;17(1):17. doi: 10.1186/s12968-015-0125-1 (PMC4331385; doi:10.1186/s12968-015-0125-1)
Supplement: Additional file 1: — Mean microvascular characteristics (SD) estimates for healthy volunteers and for all 3 invasive coronary angiography/fractional flow reserve Groups. [file 12968_2015_125_MOESM1_ESM.docx]

|  | DP    Dual bolus | DP    Single bolus | DP-First pass  Dual bolus | DP-First pass    Single bolus | Group 1 | Group 2 | Group 3 |
| --- | --- | --- | --- | --- | --- | --- | --- |
| Permeability  Surface area  product-Stress  (mL/min/mL) | 0.98 (0.32) | 1.09 (0.21) | 1.09 (0.33) | 1.21 (0.24) | 0.95 (0.18) | 0.61 (0.20) | 0.50 (0.20) |
| Permeability  Surface area  product-Rest  (mL/min/mL) | 0.56 (0.15) | 0.62 (0.14) | 0.57 (0.12) | 0.64 (0.13) | 0.53 (0.10) | 0.58 (0.10) | 0.59 (0.14) |
| Extraction fraction-  Stress (%) | 0.45 (0.04) | 0.45 (0.03) | 0.46 (0.09) | 0.48 (0.03) | 0.47 (0.04) | 0.50 (0.05) | 0.52 (0.06) |
| Extraction fraction-  Rest (%) | 0.56 (0.04) | 0.54 (0.03) | 0.58 (0.01) | 0.58 (0.02) | 0.58 (0.02) | 0.52 (0.04) | 0.53 (0.05) |
| Intravascular space-  Stress (%) | 0.08 (0.02) | 0.09 (0.02) | 0.07 (0.02) | 0.07 (0.02) | 0.07 (0.01) | 0.07 (0.03) | 0.05 (0.03) |
| Intravascular space-  Rest (%) | 0.04 (0.01) | 0.04 (0.01) | 0.03 (0.01) | 0.03 (0.01) | 0.03 (0.01) | 0.04 (0.02) | 0.04 (0.02) |
| Extravascular-  extracellular  space-Stress (%) | 0.17 (0.05) | 0.20 (0.04) | 0.15 (0.06) | 0.16 (0.03) | 0.19 (0.03) | 0.20 (0.07) | 0.18 (0.06) |
| Extravascular-  extracellular  space-Rest (%) | 0.17 (0.06) | 0.20 (0.05) | 0.15 (0.04) | 0.16 (0.04) | 0.17 (0.04) | 0.21 (0.04) | 0.23 (0.05) |
| Distribution  volume-Stress (%) | 0.22 (0.03) | 0.25 (0.03) | 0.16 (0.04) | 0.18 (0.04) | 0.21 (0.04) | 0.24 (0.05) | 0.21 (0.05) |
| Distribution volume-Rest (%) | 0.20 (0.04) | 0.23 (0.02) | 0.15 (0.04) | 0.17 (0.04) | 0.19 (0.04) | 0.22 (0.05) | 0.20 (0.05) |

Additional file 1) Mean microvascular characteristics (SD) estimates for healthy volunteers and for all 3 invasive coronary angiography/fractional flow reserve Groups.
